# Lean Breed Landrace Pigs Harbor Fecal Methanogens at Higher Diversity and Density than Obese Breed Erhualian Pigs

**DOI:** 10.1155/2012/605289

**Published:** 2012-07-16

**Authors:** Yu-heng Luo, Yong Su, André-Denis G. Wright, Ling-li Zhang, Hauke Smidt, Wei-yun Zhu

**Affiliations:** ^1^Laboratory of Gastrointestinal Microbiology, College of Animal Science and Technology, Nanjing Agricultural University, Nanjing 210095, China; ^2^Laboratory of Microbiology, Wageningen University, Dreijenplein 10, 6703 HB Wageningen, The Netherlands; ^3^Department of Animal Science, University of Vermont, 570 Main Street, Burlington, VT 05405, USA

## Abstract

The diversity of fecal methanogens of Erhualian (obese type) and Landrace (lean type) pigs was examined using separate 16S rRNA gene libraries for each breed. A total of 763 clones were analyzed; 381 from the Erhualian library and 382 from the Landrace library were identified belonging to the genus Methanobrevibacter. Others were identified belonging to the genus Methanosphaera. The two libraries showed significant differences in diversity (*P* < 0.05) and composition (*P* < 0.0001). Only two operational taxonomic units (OTUs) were found in both libraries, whereas six OTUs were found only in the Erhualian library and 23 OTUs were found only in the Landrace library. Real-time PCR showed that the abundance of fecal methanogens in Landrace pigs was significantly higher than that in Erhualian pigs (*P* < 0.05). Results showed that the Landrace pig (lean) harbored a greater diversity and higher numbers of methanogen *mcrA* gene copies than the Erhualian pig (obese). These differences may be related to the fatness or leanness in these two pig breeds. The results provide new leads for further investigations on the fat storage of pigs or even humans.

## 1. Introduction

Methanogenic archaea exist widely in the gastrointestinal (GI) tract of many vertebrates and invertebrates including humans [1–3]. Methanogens can use hydrogen and other products such as formate, methanol, acetate to reduce carbon dioxide to methane. Methane formation not only contributes to global warming as a greenhouse gas, but also represents an energy loss for the animal.

Pigs were estimated to typically lose 1.2% of ingested energy due to methane formation [4]. Furthermore, it was shown in a germ-free mouse model that methanogens play an important role in energy metabolism and adipose deposition through the re-colonization or co-colonization of the human fecal isolate *Methanobrevibacter smithii* and *Bacteroides thetaiotaomicron* into the GI tract of mice [5, 6]. Further research in humans showed that the diversity of *Mbb. smithii* concentration was higher in anorexic patients than in a lean population [7]. It was also reported that the GI tract microbiota from obese individuals was depleted in *Mbb. smithii* [8].

Archaea in the human GI tract comprise mainly members of the order *Methanobacteriales*, which are H_2_-oxidizing methanogens. Interestingly, the number of H_2_-utilizing methanogens was significantly higher in obese individuals than in lean or postgastric-bypass individuals [9]. These reports may suggest some relationship between the composition and abundance of GI tract methanogenic communities, and the host's energy metabolism, which subsequently relates to the fatness or leanness of the host.

Pigs share a high similarity with humans with respect to the anatomy, physiology and metabolism of the digestive system [10]. Thus, the composition of GI tract microbiota of obese and lean pigs could reflect that of corresponding human phenotypes. Erhualian and Landrace breeds are typically obese and lean pigs [11–14], respectively, thus their energy metabolism might be distinctive. Erhualian pig, a sister breed to Meishan (both belonging to Taihu pig), is a local porcine breed mainly located around the Taihu Lake area of China and is characterized by increased fat storage, tasty meat quality and high fertility [15]. In contrast, Landrace is an “alien” breed, which is usually used as a sire in the breeding of commercial pork production for the breed's high growth rate and lean meat percentage. According to previous research described above, the distinction between the two breeds may be partially contributed by their different gut microbiota. Recently, it has been found that obese Meishan pigs showed an increased relative abundance of *Firmicutes* and lower numbers of *Bacteroidetes *[16], in line with several reports from humans as described above. Although the diversity and abundance of bacteria between obese and lean pigs have been shown to vary [12, 16], there is no evidence has been shown regarding the variation of intestinal methanogens between the two phenotypes. Considering the key role of gut methanogens during the microbial fermentation, we hypothesized that the two breeds will have different composition and density of GI tract methanogens.

Therefore, this study investigated the phylogenetic diversity and community structure of methanogens in the feces from Erhualian (obese) and Landrace (lean) pigs by analyzing 16S rRNA gene sequences from two clone libraries, one for each breed. In addition, the density of methanogens was quantified by real-time PCR targeting the *mcrA* gene. The results shown here could provide new leads towards understanding and control of the role of GI tract microbiota in fat storage of pigs and potentially humans.

## 2. Material and Methods

### 2.1. Sample Sources and Processing

All Erhualian and Landrace pigs were raised at a commercial farm in Jiangsu Province using the same feed and under the same environmental conditions. Piglets were weaned 45 days after birth. Fecal samples from 3 suckling (40 d) piglets, 4 weaned (50 d) pigs, 4 growing (70 d) pigs, and 4 sows (11 to 12 months) of Erhualian breed, and 4 suckling piglets, 3 weaned pigs, 3 growing pigs, and 4 sows of Landrace breed were collected. Animals were randomly selected from different litters. Approximately 10 g of feces from each pig was collected into a sterilized 15 mL centrifuge tube and stored at −20°C until further processing.

### 2.2. DNA Extraction, PCR Amplification, and Clone Library Construction

Nucleic acids were extracted from 0.5 g of fecal material, based on the bead-beating method described by Zoetendal et al. [17]. The extracted DNA was purified with a PCR Clean-Up system (Promega, USA) and stored at −20°C.

Primers Met86F and Met1340R were used to amplify archaeal 16S rRNA genes [18]. The amplification was initiated with a denaturation at 94°C for 3 min, then followed by 40 cycles of 94°C for 30 s, 58°C for 30 s, 72°C for 90 s, and a final extension at 72°C for 10 min. The PCR reaction mixture (50 *μ*L) consisted of 200 nM of each primer, approximately 0.35 *μ*g of template DNA, 1× *Taq* reaction buffer, 200 *μ*M of each dNTP, 2 mM of MgCl_2_ and four units of *Taq* DNA polymerase. The product was purified using a PCR Clean-Up system (Promega, USA).

To construct 16S rRNA gene clone libraries, equal quantities of purified PCR products from animals of same breed (i.e., Erhualian, Landrace) were pooled. Cloning of pooled amplicons into *Escherichia coli* TOP10 using the pGEM-T Easy vector (Promega, USA), and screening of transformants using RFLP analysis of the cloned 16S rRNA genes by restriction digestion with endonucleases *Hae* III, *Alu* I and *Hpa* II, was done as described previously [19]. Clones with identical RFLP patterns were defined as one phylotype. One representative clone from each RFLP pattern was sequenced in both directions commercially (Invitrogen, China).

### 2.3. Estimation of Archaeal Diversity and Phylogenetic Analysis

Based on a species-level sequence identity criterion of 98% [20], MOTHUR [21] was used to assign sequences across the two libraries to operational taxonomic units (OTUs). As part of the MOTHUR suite of programs, Shannon Index was used to analyze diversity and Libshuff analysis was used to compare population structure between the two libraries. The sampling effort in each library for species-level OTUs was evaluated by calculating the coverage (*C*) according to the equation *C* = 1 − (*n*/*N*), where *n* is the number of OTUs represented by a single clone and *N* is the total number of clones analyzed in the library [22]. GenBank's BLAST program [23] was used to presumptively identify the nearest validly described neighbor of each methanogen sequence. Lastly, a neighbor-joining tree was constructed using the phylogenetic software PHYLIP (ver 3.69) with 1,000 bootstrap resamplings [24].

### 2.4. Quantification of Total Fecal Methanogens of Each Breed by Real-Time PCR

The abundance of fecal methanogens was determined with real-time PCR using an Applied Biosystems 7300HT Real-Time PCR System (Applied Biosystems, CA, USA). Primers targeting the *mcrA *gene [25] were used for the specific detection of methanogenic archaea. DNA samples extracted from each fecal sample of each breed (total 15 Erhualian pigs and 14 Landrace pigs) as described above were used for the real-time PCR amplification of *mcrA* gene. DNA from cells of a pure culture of *Methanobrevibacter smithii* supplied by CSIRO Livestock Industry (Brisbane, Australia) was also extracted with a Genomic DNA Purification Kit (Promega, USA). The concentrations of the above DNA samples extracted from fecal materials, or from cells of the pure culture, were determined in triplicate with a NanoDrop ND-1000 UV Spectrophotometer (NanoDrop Technologies, USA) and the mean values were calculated. Serial dilutions of DNA extracted from *Methanobrevibacter smithii* cells were used to generate a standard curve. A reaction mixture (10 *μ*L) consisted of 5 *μ*L of IQ SYBR Green Supermix (Roche, Basel, Switzerland), 0.5 *μ*L of each primer (10 *μ*M), and 1 *μ*L of template DNA (100 ng/*μ*L). PCR was performed with an initial denaturation step of 94°C for 2 min, followed by 30 cycles of 94°C for 30 s, 60°C for 15 s and 68°C for 1 min.

Differences in the abundance of total fecal methanogens between Erhualian and Landrace pigs were tested for significance with an One-Sample *t*-test method using the statistical software SPSS 16.0. Differences were considered significant when *P* < 0.05.

### 2.5. Nucleotide Sequences and Accession Numbers

Phylotypes were designated by using the prefix LGM (Laboratory of Gastrointestinal Microbiology) followed by either “Er” or “La” to represent the two pig breeds, Erhualian and Landrace, respectively; a number to indicate the unique phylotype (e.g., phylotype 7 from the Erhualian pig breed is LGM-Er7).

The nucleotide sequences reported in this paper have been deposited in the GenBank database under accession numbers. HM573393 to HM573406 (Erhualian) and HM573407 to HM573449 (Landrace).

## 3. Results

### 3.1. The Density of Total *mcrA* Gene Copies in the Feces of the Two Breed Pigs

Quantitative real-time PCR showed that the number of *mcrA* gene copies in the feces of Landrace pigs 8.80 ± 0.91 (Log10 (*mcrA* gene copies per gram of wet weight)) was significantly higher than that in the Erhualian pigs (8.23 ± 0.63, *P* < 0.05).

### 3.2. Sequence Analysis of the Two Archaeal 16S rRNA Gene Clone Libraries

**Figure 1:**
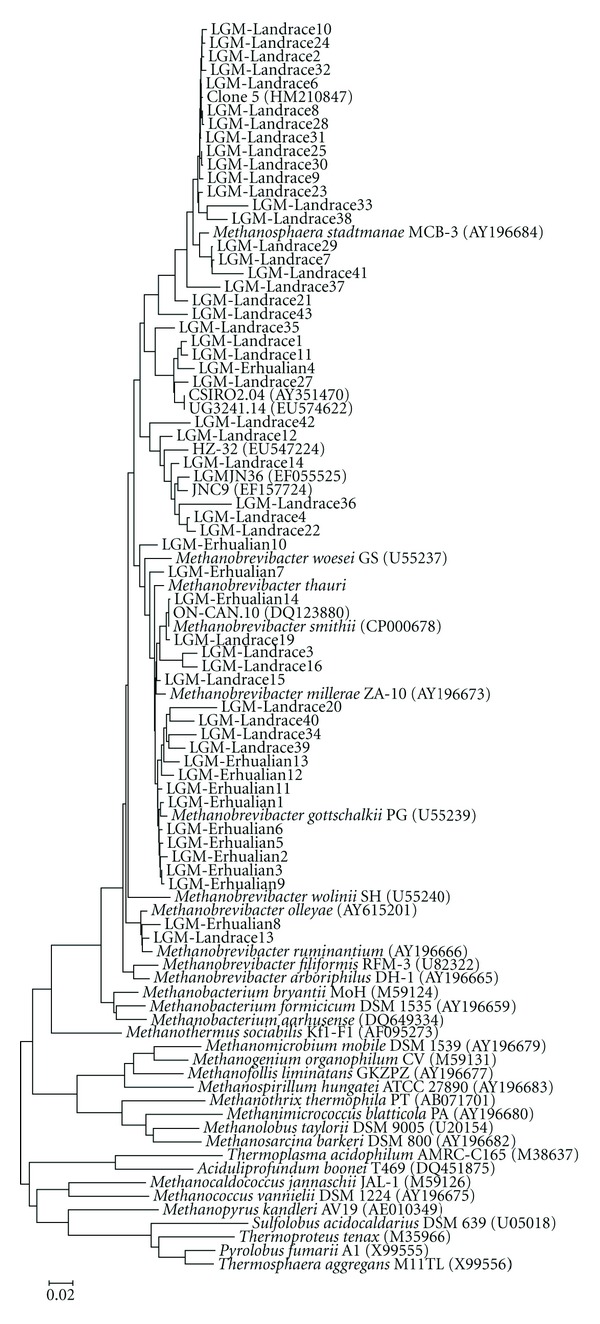
Phylogenetic relationship of archaeal 16S rRNA gene sequences retrieved from fecal samples of Erhualian and Landrace pigs. Evolutionary distances were calculated using the Neighbor-Joining method. The tree was bootstrap resampled 1000 times.

A total of 381 cloned archaeal 16S rRNA gene amplicons, obtained from fecal samples taken from Erhualian pigs at different life stages, were analyzed. Sequence examination of these clones revealed eight different OTUs (Table 1). The majority of sequences (368/381) were most closely related to members belonging to the genus *Methanobrevibacter* with sequence identities ranging from 96.9% to 99.9%. One hundred and forty-one sequences (37%) were assigned to OTU11 (Table 2) and related to *Methanobrevibacter gottschalkii* and *Methanobrevibacter millerae*, while 111 sequences (29%) were assigned to OTU13 and related to *Methanobrevibacter smithii *(Table 2). Clone LGM-Er7 (OTU2) was distantly related to *Methanobrevibacter millerae* with 96.9% identity, but had 97.7% identity to uncharacterized *Methanobrevibacter* clones from the foregut of the Tammar wallaby [26]. LGM-Er8 (OTU3) was related to *Methanobrevibacter ruminantium* (97.1% identity), but had 99.8% identity to clones from a coculture with anaerobic fungi (Cheng et al., unpublished data). Only clone LGM-Er4 (OTU1) was distantly related to *Methanosphaera cuniculi* (96.3% identity). OTUs 1, 2, 3, 4, 5, and 6 were unique to the Erhualian library (Tables 1 and 2; Figure 1).

The 16S rRNA gene library from Landrace pigs was comprised a total of 382 clones, consisting of 25 OTUs (Table 1). Most of sequences (227/382) were related to members belonging to the genus *Methanobrevibacter* with sequence identities ranging from 93.9% to 99.5%. Eighty-nine sequences (23%) were assigned to OTU13 and related to *Methanobrevibacter smithii*, while 81 sequences (21%) were assigned to OTU20 and related to *Methanosphaera cuniculi* (Tables 1 and 2). LGM-La2 (OTU20), LGM-La23 (OTU20), and LGM-La38 (OTU26) showed 97.6%, 97.4%, and 97.4% identity to *Methanosphaera cuniculi*, respectively, but had even higher (99.8%, 99.6%, and 99.1%) sequence identities to uncharacterized clones from pig feces [27]. Although LGM-La3 (OTU7) had 95.3% identity to *Methanobrevibacter millerae* it had 97% identity to unidentified clones from the Holstein and Jersey dairy cows in the USA [28]. LGM-La29 (OTU19) showed 97.1% to *Methanosphaera stadtmanae* and had 97.4% to sequences from rumen of goats (Pei unpublished data). LGM-La33 (OTU21) was distantly related to *Methanosphaera cuniculi* with 95.5% identity, but had 97.1% identity to clones from pig feces [27]. LGM-La40 was found 96.8% identity related to *Methanobrevibacter smithii*, but had (OTU28) 98.2% identity to clones from the rumen of Norwegian reindeer [29]. LGM-La1 (OTU17), LGM-La27 (OTU18), LGM-La35 (OTU23), and LGM-La11 (OTU17) were related to *Methanobrevibacter smithii *and *Methanosphaera cuniculi* with 93.8%–94.9% identity, but had 95.7%–96.8% identity to clones from lactating dairy cows in Canada [30]. LGM-La21 (OTU15), LGM-La36 (OTU24), and LGM-La37 (OTU25) were related to *Methanosphaera cuniculi* with 94.2%–95.5% identity, but had 95.3%–96.8% identity to clones from pig feces [27]. LGM-La12 (OTU8) and LGM-La14 (OTU10) had 93.9% and 95.1% identity, respectively, to *Methanobrevibacter smithii* and* Methanosphaera cuniculi*, but had only 96.0% and 96.9% identity to clones from the crops of *Opisthocomus hoazin*, a folivorous bird from Venezuela [20]. LGM-La4 (OTU16) and LGM-La22 (OTU16) showed 95.5% and 95.7% identity related to *Methanosphaera stadtmanae,* but had 96.8% and 96.9% identity to clones from Jinnan cattle (Pei et al., unpublished data). LGM-La42 (OTU30) and LGM-La43 (OTU31) were distantly related to *Methanosphaera cuniculi* and *Methanobrevibacter smithii* with 95.3% identity, respectively, but had only 96.3% and 96.2% to their nearest neighbor, clones from the Holstein and Jersey dairy cows [28]. It is important to note that OTUs except OTU11 and OTU13 were only found in this library (Table 1, Figure 1).

## 4. Discussion

Anecdotal evidence from a limited number of previous studies indicated that methanogens in the hindgut of monogastric animals may play a critical role in their host's energy metabolism and adipose deposition as revealed by inoculating human-derived methanogens into a germ free mouse model [5]. However, due to its particular growth environment and the intricate syntrophic interactions with other intestinal bacteria, culture-dependent methods provide limited information on methanogens in the gut. Numerous studies using culture-independent methods including 16S rRNA gene clone library analysis, have reported data on methanogen diversity and abundance in the rumen [19, 25, 31, 32]. However, very little information is available on the diversity and abundance of methanogenic archaea in the gut of monogastric animals including humans and pigs. The present study used Erhualian and Landrace pigs as surrogate models for obese and lean host phenotypes and provided the first account on the comparison of methanogen diversity and abundance in feces of host species with either an obese or lean phenotype. Fecal samples were collected from each breed pigs at four different ages to make the result more representative, as it was expected to cover the whole growth stages of each breed.

Real-time PCR results showed that Landrace pigs had significantly more *mcrA* gene copies than Erhualian pigs (*P* < 0.05), which suggests that there are more numbers of methanogens harboring in the hindgut of Landarce pigs. Moreover, Landrace pigs exhibited significantly more methanogen diversity (*P* < 0.05) than Erhualian pigs (Table 3). Libshuff analysis also indicated that differences in the community structure between the two libraries were significant (*P* < 0.0001). Clone library OTU coverages were estimated at 100% and 99.2% for Erhualian and Landrace pigs, respectively (Table 3). This indicates that Landrace pigs, which are recognized for their higher lean meat proportion and lower body fat mass as compared to Erhualian pigs, harbored more colonic methanogens (density and diversity). Methanogens can produce methane from substrates such as H_2_ and CO_2_ and formate, which could also be used for the formation of propionate and acetate. For ruminants, it is widely established that the formation of methane results in a loss of energy available for the host [33, 34]. Thus, a highly dense and diverse methanogen community as observed in fecal samples may also suggest an energy loss, which may consequently affect energy metabolism and body fat mass formation. It is also possible that although methane formation may represent a small portion of energy for a pig growth, it may affect metabolic pathway network and consequently affect the energy metabolism.

In total, 763 clones were examined from the two 16S rRNA gene clone libraries, revealing 53 phylotypes assigned to 31 OTUs (Table 1). OTUs 11 and 13 were the only OTUs found in both pig breeds (Table 2) and accounted for 66% and 24% of the clones from Erhualian and Landrace pigs, respectively. Interestingly, clones belonging to OTU11 were nearly 35 times higher in Erhualian library than Landarce library. OTUs 11 and 13 combined for 70% of all OTUs in Erhualian library, but only 36% of all OTUs in the Landrace library (Table 2).

The comparison of OTUs between the two libraries (Table 2) showed that *Methanobrevibacter*-like sequences (96.6%) were dominant in the feces of Erhualian pigs, whereas the proportion was 56.5% for Landrace pigs. Furthermore, *Methanosphaera*-like sequences accounted for 3.4% in Erhualian and 43.5% in Landrace. This is consistent with previous findings that *Methanobrevibacter *species are the predominant methanogen in the hindgut of monogastric animals [27, 35–42] or in the rumen of sheep [31, 43–45], cattle [45–48] and also in some cultivation studies [49]. Nevertheless, in the rumen of sheep and bovine, the most dominant methanogens belonged to genus *Methanobrevibacter*, and the density of *Methanosphaera*-like species was much less than that of *Methanobrevibacter*-like species [45, 46]. While in the rumen of sheep from Western Australia, *Methanosphaera stadtmanae* was only found in a minority of sheep [31]. In our previous study, the proportion of *Methanosphaera stadtmanae* 16S rRNA sequences was very small in Duroc × Landrace × Yorkshire pig feces [27]. However, in the present study, Landrace pigs had 10 times more *Methanosphaera*-like methanogens than Erhualian pigs, while *Methanosphaera*-like species were the second dominant methanogens behind *Methanobrevibacter* in the feces of both pig breeds.

Our libraries also contained several yet unidentified euryarchaeotic sequences. Fourteen OTUs (LGM-La1 and 11, 3, 4 and 22, 12, 14, 21, 27, 33, 35, 36, 37, 42, 43 and LGM-Er4) were most likely represent yet unknown methanogenic species and strains (Table 1). Interestingly, in a previous study we found that populations related to *Aciduliprofundum boonei* and *Thermoplasma acidophilum* were present, in addition to the *Methanobrevibacter* and *Methanosphaera* populations, in the feces of Duroc × Landrace × Yorkshire pigs [27]. Methanogens related to the Thermoplasmatales clade were also found in some ruminants [19, 32, 46, 50], whereas in the current study, most sequences were associated with the two genera *Methanobrevibacter* and *Methanosphaera*. This suggests that there might be a difference in the diversity of gut methanogens between pure breeds and hybrids, and hybridization of different breeds might introduce a certain alteration of the methanogenic diversity into the intestine.

## 5. Conclusion

The current study provided the first account on the abundance and phylogenetic diversity of methanogens found in Erhualian (obese) and Landrace (lean) pig feces based on 16S rRNA gene clone library analysis. Landrace pigs have a markedly higher density of methanogens than the Erhualian pigs (*P* < 0.05). The diversity of methanogens of Landrace pigs was also significantly higher than that of Erhualian animals (*P* < 0.05) with *Methanobrevibacter* as the most dominant genus in both breeds, and *Methanosphaera* being the second most dominant methanogen in Landrace pigs. The functional roles of these methanogens in the pig gut, and whether observed differences in methanogen diversity and density are related to the pig fat or energy metabolism, need further investigation.

## Figures and Tables

**Table 1:** 16S rRNA sequences from feces of Erhualian and Landrace pigs.

Erhualian			Landrace		
16S phylotypes	No. clones	OTU^#^	16S phylotypes	No. clones	OTU^#^
LGM-Er1	19	11	LGM-La1	11	17
LGM-Er2	49	11	LGM-La2	15	20
LGM-Er3	6	11	LGM-La3	5	7
LGM-Er4	13	1	LGM-La4	12	16
LGM-Er5	4	11	LGM-La6	2	20
LGM-Er6	7	11	LGM-La7	6	19
LGM-Er7	5	2	LGM-La8	25	20
LGM-Er8	81	3	LGM-La9	2	20
LGM-Er9	6	11	LGM-La10	2	20
LGM-Er10	15	4	LGM-La11	8	17
LGM-Er11	50	11	LGM-La12	6	8
LGM-Er12	6	5	LGM-La13	4	9
LGM-Er13	9	6	LGM-La14	5	10
LGM-Er14	111	13	LGM-La15	4	11
			LGM-La16	2	12
			LGM-La19	89	13
			LGM-La20	43	14
			LGM-La21	5	15
			LGM-La22	5	16
			LGM-La23	2	20
			LGM-La24	5	20
			LGM-La25	11	20
			LGM-La27	2	18
			LGM-La28	2	20
			LGM-La29	4	19
			LGM-La30	4	20
			LGM-La31	10	20
			LGM-La32	1	20
			LGM-La33	12	21
			LGM-La34	7	22
			LGM-La35	6	23
			LGM-La36	6	24
			LGM-La37	2	25
			LGM-La38	3	26
			LGM-La39	1	27
			LGM-La40	46	28
			LGM-La41	2	29
			LGM-La42	4	30
			LGM-La43	1	31

**Table 2:** Comparison of OTUs between Erhualian and Landrace pigs.

OTU	No. of sequences		Nearest valid taxon^∗^	% Seq. identity
	Erhualian	Landrace		
1	13	—	*Methanosphaera cuniculi*	96.3
2	5	—	*Methanobrevibacter millerae*	96.9
3	81	—	*Methanobrevibacter ruminantium*	97.1
4	15	—	*Methanobrevibacter ruminantium*	97.1
5	6	—	*Methanobrevibacter gottschalkii*	97.4
6	9	—	*Methanobrevibacter millerae*	97.8
7	—	5	*Methanobrevibacter millerae*	95.3
8	—	6	*Methanobrevibacter smithii*	93.9
9	—	4	*Methanobrevibacter ruminantium*	98.8
10	—	5	*Methanosphaera cuniculi*	95.1
11	141	4	*Methanobrevibacter gottschalkii*	98.8
12	—	2	*Methanobrevibacter smithii*	97.0
13	111	89	*Methanobrevibacter smithii*	99.9
14	—	43	*Methanobrevibacter smithii*	97.1
15	—	5	*Methanosphaera cuniculi*	94.6
16	—	17	*Methanosphaera stadtmanae*	95.7
17	—	19	*Methanosphaera cuniculi*	94.9
18	—	2	*Methanobrevibacter smithii*	94.2
19	—	10	*Methanosphaera stadtmanae*	97.2
20	—	81	*Methanosphaera cuniculi*	98.1
21	—	12	*Methanosphaera cuniculi*	95.5
22	—	7	*Methanobrevibacter millerae*	97.4
23	—	6	*Methanobrevibacter smithii*	94.5
24	—	6	*Methanosphaera cuniculi*	94.2
25	—	2	*Methanosphaera cuniculi*	95.5
26	—	3	*Methanosphaera cuniculi*	97.4
27	—	1	*Methanobrevibacter millerae*	98.5
28	—	46	*Methanobrevibacter smithii*	96.8
29	—	2	*Methanosphaera stadtmanae*	96.7
30	—	4	*Methanosphaera cuniculi*	95.3
31	—	1	*Methanobrevibacter smithii*	95.3

Totals	381	382		

^∗^Nearest valid taxon is represented by the type strain of the designated species.

**Table 3:** Coverage and Shannon Index calculated using MOTHUR^1^ for each methanogen 16S rRNA gene clone library.

Clone library	OTUs observed	CHAO 1 OTU estimate	% OTU coverage^2^	Shannon index ± 95% confidence limits	Libshuff analysis
Erhualian	8	8	100	1.51 ± 0.08^a^	*P* < 0.0001
Landrace	24	24.2	99.2	2.38 ± 0.11^b^	*P* < 0.0001

^1^Schloss et al. [21].

^2^Good's [22] coverage (*C*) according to the equation *C* = 1 − (*n*/*N*), where *n* is the number of sequences represented by a single clone and *N* is the total number of clones in the library.

^a,b^There is significant difference between these values.
